# The effects of green tea tablets and metformin on ovulation and menstrual cycle regularity in women with polycystic ovary syndrome

**DOI:** 10.25122/jml-2022-0066

**Published:** 2024-01

**Authors:** Mahnaz Yavangi, Soghra Rabiee, Marzieh Sanavi Farimani, Shahede Khansary, Maryam Farhadian, Akram Ranjbar, Minoo Mahmoudi, Masoud Karimi, Somayeh Barati, Amir Barati Mosleh, Noushin Mohammadpour

**Affiliations:** 1 Department of Gynecology, School of Medicine, Hamadan University of Medical Sciences, Hamadan, Iran; 2 Department of Biostatistics, School of Health, Hamadan University of Medical Sciences, Hamadan, Iran

**Keywords:** green tea, metformin, polycystic ovary syndrome

## Abstract

Polycystic ovary syndrome is the most common cause of oligo-ovulation and anovulation among women of reproductive age, contributing to infertility. This study aimed to compare the effects of green tea tablets and metformin on ovulation, menstrual cycle regularity, and antioxidant biomarkers in women with polycystic ovary syndrome (PCOS). In this clinical trial study, 94 women with PCOS were randomly assigned to three groups: green tea (*n* = 33), metformin (*n* = 29), and control (*n* = 32). Menstrual status and oxidative stress parameters, including total antioxidant capacity, thiol, and lipid peroxidation, were compared before and 3 months after the intervention among all three groups. Data analysis was conducted using SPSS software version 22 and employing the analysis of variance and paired t-tests. Following the intervention, the mean menstrual cycle duration in the green tea, metformin, and control groups was 32.22 ± 12.78, 48.72 ± 37.06, and 48.53 ± 31.04 days, respectively (*P* = 0.040). There was no statistically significant difference between the three groups in terms of biochemical, hormonal, and antioxidant indices before and after the intervention (*P* > 0.05). The intake of green tea tablets was associated with better outcomes in regulating the menstrual cycle in women with PCOS.

## INTRODUCTION

Polycystic ovary syndrome (PCOS) is one of the most common endocrine disorders, with reported prevalence rates varying between 4% and 25% worldwide, depending on the specific diagnostic criteria used [1-3]. Patients with PCOS often experience conditions such as depression, low self-esteem, anxiety, and several metabolic disorders, in addition to increased reproductive problems, including infertility, endometrial cancer, late-onset menopause, and ovarian diseases [4-7]. The cause of PCOS is not completely clear; however, the most common causes are genetic predisposition, increased insulin secretion, insulin resistance, obesity, and environmental and chemical contamination [4,8,9]. The rate of early miscarriages during the first trimester in women with PCOS is three times higher than in women without the syndrome [10,11]. Excess weight accelerates the onset of clinical manifestations in women prone to this syndrome [12].

The accumulation of visceral fat in patients with PCOS contributes to the production of inflammatory cytokines, which can exacerbate insulin resistance—a key factor in the pathogenesis of PCOS [13]. Insulin directly stimulates the ovaries to produce estradiol and androgens, strengthening the activity of luteinizing hormone (LH) and follicle-stimulating hormone (FSH). It also increases ovarian aromatase activity, facilitating the conversion of androgens to estrogens [14,15]. Patients with PCOS have elevated levels of plasma leptin [16,17], a hormone considered the link between nutrition and the reproductive system [18] and which is involved in the development of PCOS [19]. Serum leptin levels are significantly correlated with body mass index (BMI) and insulin resistance [20]. Both insulin resistance and BMI play a key role in the context of PCOS [13,21,22]. Leptin also increases LH levels by suppressing neuropeptide Y in the hypothalamus, highlighting the complex interplay of insulin resistance, BMI, and hormonal imbalance in PCOS [23].

Weight reduction leads to decreased leptin concentrations, which consequently increases activity in brain regions responsible for regulating food intake, encompassing emotional, cognitive, and sensory processes. This suggests that maintaining leptin levels stable during weight loss, possibly through hormone replacement therapy, might override the homeostatic and behavioral tendencies toward energy conservation and weight regain during dieting [24].

Serum concentrations of testosterone and androstenedione are higher in women with PCOS [25,26]. Given the important role of insulin resistance in PCOS pathogenesis, medications aimed at lowering insulin levels have emerged as potential treatments, with metformin being the most commonly prescribed drug [27]. Metformin has shown positive effects on ovulation in patients with PCOS [28], reducing LH levels, free LH, free radicals, FSH, and sex hormone-binding globulin (SHBG) in women with obesity and PCOS [29]. The beneficial effect of green tea on obesity in patients with PCOS has been previously shown [30], although more evidence is needed.

The increase in insulin resistance in women with PCOS may be attributed to their elevated BMI and LH levels [31]. Clinical manifestations of PCOS, such as anovulation, infertility, and obesity, involve dysfunction in the hypothalamic-pituitary axis, ovarian function, and insulin activity [32,33]. This study aimed to compare the effects of green tea tablets and metformin on ovulation, menstrual cycle regularity, and antioxidant biomarkers in women diagnosed with PCOS.

## MATERIAL AND METHODS

### Study design and setting

This randomized clinical trial included 94 women of reproductive age diagnosed with PCOS, with clinical signs of insulin resistance and a BMI ≥ 25. Participants were recruited using convenience sampling from those referred to Fatemieh Hospital in 2021, provided they fulfilled the specified inclusion criteria for the study.

### Eligibility criteria

Inclusion criteria comprised patients with PCOS of reproductive age, presenting clinical symptoms of insulin resistance and a BMI ≥25, oligoovulation, multiple ovarian cysts (follicles with a diameter of 2 to 6 mm), and irregular periods compared to individuals without PCOS. Exclusion criteria included patients who were not willing to continue treatment or failed to attend scheduled treatment visits, those with heart or renal failure, hyperthyroidism, mental conditions such as anxiety, individuals with a sensitive stomach or caffeine sensitivity, and those who experienced gastrointestinal side effects during a pregnancy test or as a result of taking metformin. Additionally, individuals treated with warfarin, aspirin, propranolol, and alkaline drugs were also excluded.

### Randomization and blinding

The patients were allocated to the study groups using balanced block randomization with a block size of 6. The patients were unaware of the type of intervention, making this a single-blind trial.

### Intervention

After obtaining written informed consent, participants were randomly assigned to three groups. The first group received 500 mg of green tea (500 mg of green tea leaf powder enriched with a standardized extract containing 50 mg of total polyphenol, Dineh Company) twice a day for one week and then 1500 mg daily for three months. The second group received metformin (500 mg metformin, Aria Company) daily for three months. The third group (control) received the same quantity of placebo (500 mg pills with an inactive filter) for three months.

### Study procedure

Blood samples (5 cc) were collected from all patients before the start of treatment and after the intervention to measure oxidative stress markers and antioxidant capacity. Oxidative stress parameters included total antioxidant capacity measured by the Ferric Reducing Ability of Plasma (FRAP) method [34], thiol group levels measured by the HU method [35], and lipid peroxidation measured by the Satho method [36]. All experiments were performed under standard conditions and according to the instructions of the manufacturer in the toxicology laboratory of Hamadan School of Pharmacy. The BMI of the patients was measured by dividing their weight in kilograms by the square of their height in meters. Blood pressure measurements were extracted from the medical files. Ultrasound scans were performed to determine endometrial and ovarian thickness and follicle size. Waist circumference was determined by placing a tape measure horizontally around the abdomen at the level of the iliac crest, with measurements taken twice and then averaged. Similarly, hip circumference was measured by encircling the widest part of the buttocks with a tape measure, again measured twice and averaged.

### Statistical analysis

Data were collected according to the research objectives and analyzed using SPSS 21 statistical software. We used one-way analysis of variance (ANOVA) and paired t-tests for within-group comparisons. The significance level was set at less than 0.05.

## RESULTS

**Figure 1:**
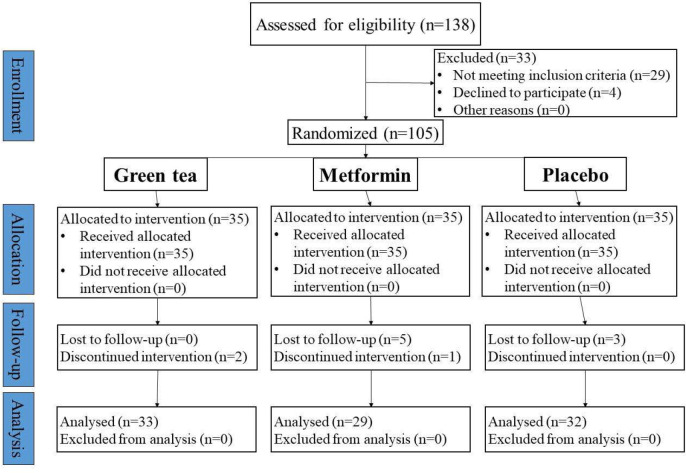
Flowchart of the patients in the clinical trial

Figure 1 shows the CONSORT flow chart. In total, 138 patients were enrolled in the study. Among them, 105 individuals met the eligibility criteria and were subsequently divided into three groups using balanced block randomization, with each group comprising 33 patients. Later, three patients in the placebo group and five in the metformin group were withdrawn from follow-up. One patient in the metformin group and two in the green tea group discontinued the intervention. Finally, 33 patients in the green tea group, 29 in the metformin group, and 32 in the placebo group entered the final analysis.

**Figure 2:**
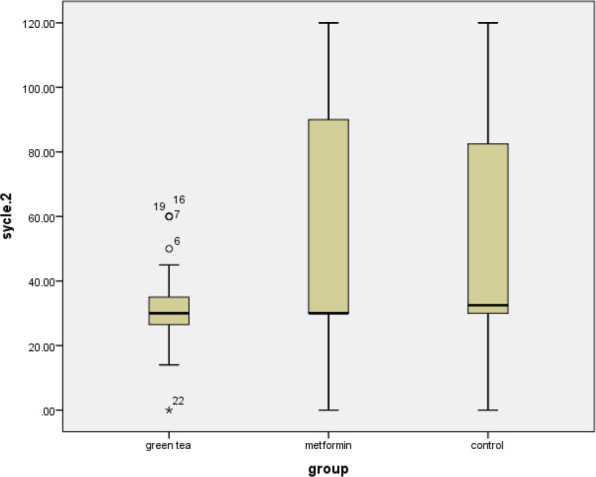
Impact of green tea and metformin on menstrual cycle

The mean age of patients in the green tea, metformin, and control groups was 27.3 ± 4.11, 27.75 ± 4.23, and 27.06 ± 4.18 years (*P* = 0.808), respectively. BMI, endometrial thickness, ovarian thickness, follicle, menstrual duration, cycle, blood pressure, and anthropometric indices were similar and were not statistically different (*P* > 0.05). BMI, waist circumference, hip measurements, and blood pressure in the green tea group showed a greater reduction compared to the other groups, although these changes were not statistically significant (*P* > 0.05) (Table 1). The only significant difference between the groups was the reduction in the interval between menstruation, which was significantly lower in the green tea group than the other two groups (*P* = 0.040) (Figure 2). There was no statistically significant difference between the three groups in terms of biochemical, hormonal, and antioxidant indices before and after the intervention (*P* >0.05). Leptin levels in the green tea group were lower than in the other groups. The mean blood glucose level was the lowest in the metformin group. At the end of the study, individuals who consumed green tea showed a significant increase in insulin and FSH levels compared to their baseline values (Table 2).

**Table 1:** Changes in anthropometric measurements and menstrual cycle duration pre- and post-intervention across treatment groups

Variable	Treatment group	Pre-intervention		Post-intervention	
		Mean ± SD	P value*	Mean ± SD	*P* value*
BMI (kg/m^2^)	Green tea	30.08 ± 3.69	0.475	27.57 ± 7.88	0.154
	Metformin	29.65 ± 3.83		29.33 ± 3.78	
	Control	30.95 ± 5.08		30.62 ± 5.13	
Endometrium thickness (mm)	Green tea	7.51 ± 3.32	0.735	7.29 ± 3.31	0.222
	Metformin	6.94 ± 2.22		8.77 ± 3.81	
	Control	7.23 ± 2.83		7.37 ± 2.65	
Ovarian follicle (mm)	Green tea	4.24 ± 1.75	0.533	4.70 ± 4.70	0.161
	Metformin	4.31 ± 2.33		4.27 ± 1.71	
	Control	4.81 ± 2.54		5.85 ± 3.94	
Waist circumference (cm)	Green tea	91.45 ± 8.75	0.339	90.19 ± 8.11	0.398
	Metformin	89.41 ± 7.71		90.54 ± 7.07	
	Control	92.81 ± 10.23		93.14 ± 10.67	
Hip circumference (cm)	Green tea	108.15 ± 7.67	0.103	108.41 ± 7.38	0.103
	Metformin	107.65 ± 5.91		108.45 ± 5.11	
	Control	111.84 ± 10.72		112.21 ± 10.01	
Systolic blood pressure (mm/Hg)	Green tea	11.48 ± 1.19	0.050	12.00 ± 1.08	0.363
	Metformin	11.00 ± 0.96		11.20 ± 1.00	
	Control	11.76 ± 1.27		15.12 ± 17.84	
Follicle (mm)	Green tea	0.09 ± 0.29	0.332	0.18 ± 0.39	0.741
	Metformin	0.13 ± 0.35		0.13 ± 0.35	
	Control	0.03 ± 0.17		0.11 ± 0.32	
Menstrual period (days)	Green tea	7.45 ± 2.52	0.637	8.36 ± 4.61	0.313
	Metformin	6.68 ± 2.73		7.09 ± 2.50	
	Control	7.28 ± 4.27		6.84 ± 4.07	
Menstrual cycle (day)	Green tea	55.60 ± 40.92	0.31	32.22 ± 12.78	0.040
	Metformin	55.52 ± 39.41		48.72 ± 37.06	
	Control	60.68 ± 46.45		42.34 ± 28.48	

* One-way ANOVA; SD, Standard deviation

**Table 2:** Hormonal, biochemical and antioxidant variables in treatment groups before and after intervention

Variable	Treatment group	Pre-intervention		Post-intervention	
		Mean ± SD	*P* value*	Mean ± SD	*P* value*
Leptin ng/mL (nanograms per milliliter)	Green tea	29.70 ± 13.24	0.629	28.69 ± 13.13	0.952
	Metformin	27.10 ± 8.73		29.8 ± 9.04	
	Control	27.80 ± 10.40		29.23 ± 13/53	
Insulin (pmol/ml)	Green tea	14.36 ± 9.86	0.901	19.0 ± 9.39	0.400
	Metformin	13.15 ± 8.03		15.27 ± 7.25	
	Control	13.40 ± 14.39		20.66 ± 19.96	
Glucose (mg/dL)	Green tea	94.21 ± 10.93	0.06	93.77 ± 15.74	0.201
	Metformin	88.48 ± 10.46		86.9 ± 10.39	
	Control	88.71 ± 10.32		90.5 ± 11.90	
TCA (pmol/ml)	Green tea	3.33 ± 0.58	0.999	3.25 ± 0.27	0.265
	Metformin	3.33 ± 0.41		3.33 ± 0.32	
	Control	3.33 ± 0.41		3.50 ± 0.89	
MDA (pmol/ml)	Green tea	9.37 ± 3.75	0.271	9.93 ± 10.44	0.401
	Metformin	8.45 ± 2.98		8.28 ± 3.81	
	Control	8.15 ± 2.40		7.40 ± 2.44	
TG (mg/dL)	Green tea	0.23 ± 0.09	0.883	0.22 ± 0.11	0.294
	Metformin	0.24 ± 0.15		0.27 ± 0.21	
	Control	0.22 ± 0.14		0.20 ± 0.09	

* One-way ANOVA

SD, Standard deviation; TCA, Tricarboxylic Acid; MDA, Malondialdehyde; TG, Triglycerides

## Discussion

The findings of the present study showed that patients who consumed green tea had lower serum leptin levels and BMI, although the differences between the groups were not statistically significant. The possible reason may be the relatively short length of the study and the limited period of medication intake among patients. However, this finding could be clinically significant. Leptin, characterized by a 16 kDa secretory protein, is produced by adipose tissue, and its levels increase with higher BMI. It acts primarily through its specific receptors in the hypothalamus, regulating appetite and energy in the body. Leptin plays a vital role in regulating a wide range of biological responses, including energy homeostasis, hematopoiesis, neuronal function, and immune responses. It performs biological reactions by activating its cognitive receptors, which belong to the class 1 cytokine receptor family [37]. Furthermore, in addition to being a hypothalamic modulator of food intake, body weight, and fat storage, leptin has a dual function in inflammation. It triggers the activation of monocytes and macrophages, producing proinflammatory cytokines such as tumor necrosis factor (TNF), interleukin-6 (IL-6), and IL-9, leading to T cell differentiation to the Th1 phenotype. Leptin has also been observed to stimulate keratinocyte proliferation, enhance the expression of adhesion molecules, and facilitate angiogenesis. Leptin and adiponectin appear to act as opposites in their metabolic functions [38].

Contrary to the findings of the present study, most research shows a positive correlation between leptin concentration and BMI, suggesting an intrinsic link with obesity, where adipokine levels generally decrease. Moreover, leptin and adiponectin are involved in inflammation, as well as metabolic functions and cardiovascular diseases. The adiponectin-encoding gene is located in the same regions as the genes involved in metabolic syndrome, type 2 diabetes, and cardiovascular diseases. Leptin is suggested to be an independent predictor of future cardiovascular events, and patients with lower adiponectin levels are at risk of coronary heart disease, type 2 diabetes, hypertension, and dyslipidemia [39,40]. Numerous studies have shown that plasma leptin levels increase in people with PCOS [16,17].

Green tea consumption has been identified as a potential therapeutic approach for weight management, as shown by Amirghofran [41]. This study highlighted significant reductions in mean BMI, weight, waist circumference, and fat percentage in individuals who consumed green tea compared to their pre-intervention values. Considering the high prevalence of metabolic syndrome in women with PCOS and the correlation between reduced leptin levels, obesity, and metabolic syndrome, green tea could be considered a part of the treatment strategy [42]. Weight loss is often the initial step in managing PCOS in women, and given the favorable effects of green tea on weight loss, it can be considered a supportive therapy. This may be particularly relevant for women with PCOS who are also managing obesity. The findings in this study revealed that patients receiving metformin had a significant decrease in their mean BMI and a significantly shorter interval between menstruations compared to their pre-intervention status. Metformin is widely recognized as one of the primary medications for treating PCOS [27]. Research has demonstrated its effectiveness, particularly in improving ovulation among patients with PCOS [28]. However, one of the concerns about using drugs during pregnancy, including metformin, is their passage through the placenta, leading to adverse effects on the fetus and an increased risk of congenital anomalies [43]. Clinical trials showed that metformin use during pregnancy is not associated with complications and abnormalities in the fetus and mother [44]. In addition, animal studies have also shown that metformin does not have a detrimental effect on the fetus [45]. However, some studies have indicated that it has a teratogenic effect in animals and increases the risk of hypoglycemia in the human fetus [46]. Metformin partially crosses the placental barrier. According to most studies, metformin can be continued during pregnancy, and there is no strong evidence of its detrimental effect on the fetus [47-49]. Metformin is also secreted in very small amounts in breast milk, although this amount cannot cause significant effects on the fetus [50]. Although metformin has been used to treat diabetes for more than three decades, its exact mechanism is still unclear. Its main effect is to reduce hepatic glucose output by inhibiting glycogenesis. Furthermore, metformin increases insulin-mediated glucose uptake into peripheral tissues such as muscle and liver, especially after a meal. It has a lipolytic effect that reduces free fatty acid concentrations, thus reducing the availability of substrate for glucogenesis. Serum insulin concentrations are slightly reduced due to improved glycemic control. Metformin also reduces food intake and body weight [30,31]. In this study, the only significant difference between the groups was the reduction in the interval between menstruations, which was statistically shorter in the green tea group compared to the other two groups. In the green tea group, there were greater reductions in BMI, waist circumference, hip measurements, and blood pressure compared to the other two groups. However, these differences were not statistically significant. Furthermore, the results did not confirm the effect of green tea consumption on antioxidant biomarkers among patients. This may be due to its short-term consumption, as well as its compounds, including epigallocatechin-3-gallate, epigallocatechin, epicatechin gallate, and epicatechin [8]. Among antioxidants, epigallocatechin-3-gallate has the highest potency, with the antioxidant power being 100 times higher than that of vitamin C and 25 times higher than that of vitamin E [9].

This study showed that green tea consumption did not lower blood sugar. The studies on the effect of green tea extract on blood sugar are incomplete. In one study, green tea had no significant effect on glycemia but inhibited hyperlipidemia and improved superoxide retinal formation [51]. Other studies suggest that certain compounds in green tea may increase glucose uptake in rat adipocytes, suggesting a role in improving insulin sensitivity [52]. However, in other studies, the effect of the extract on the prevention of blood sugar and weight loss has not been shown, although its reduction effect on food absorption has been indicated [51]. Several animal and human studies have shown that green tea has anti-diabetic effects [52,53]. The present study showed that green tea consumption was associated with increased insulin. Similar to this study, other studies have also shown that green tea flavonoids have effects on insulin [54]. It should be noted that in addition to the beneficial effects of green tea, its excessive consumption can lead to adverse effects. The effects of green tea catechins can vary among individuals. For instance, epigallocatechin gallate (EGCH) can be cytotoxic, and high consumption of green tea can exert acute cytotoxicity on liver cells. Other studies determined that EGCH acts as a pro-oxidant in pancreatic beta cells [55,56]. Therefore, it is necessary to advise patients about the excessive and arbitrary consumption of green tea, especially its extracts.

The present research did not find an effect of green tea on antioxidant biomarkers in patients, possibly due to its short-term consumption. Not assessing some biochemical parameters, including FSH, LH, estradiol, and progesterone, can be considered a study limitation, thus being suggested for investigation in future studies.

## Conclusion

This study showed that green tea consumption in patients with polycystic ovary syndrome and obesity could be associated with better outcomes in regulating the menstrual cycle. However, it is advisable to conduct further research and long-term follow-up studies to understand better the implications of green tea consumption in women with this syndrome.

## Data Availability

All data generated or analyzed during this study are included in this published article. The datasets used and/or analyzed are available from the corresponding author upon reasonable request.

## Personal thanks

The authors would like to thank the Vice-Chancellor for Research and Technology, Hamadan University of Medical Sciences, for allowing us to carry out this study. We would like to thank all participants for their participation.
